# Inhibition of Adherence and Biofilm Formation of *Pseudomonas aeruginosa* by Immobilized ZnO Nanoparticles on Silicone Urinary Catheter Grafted by Gamma Irradiation

**DOI:** 10.3390/microorganisms11040913

**Published:** 2023-03-31

**Authors:** Dalia A. Elzahaby, Hala A. Farrag, Rana R. Haikal, Mohamed H. Alkordi, Nourtan F. Abdeltawab, Mohammed A. Ramadan

**Affiliations:** 1 Department of Drug Radiation Research, National Center for Radiation Research and Technology—Egyptian Atomic Energy Authority (NCRRT), Cairo 11787, Egypt; dalia.elzahaby@outlook.com (D.A.E.); hala_farrag_24@hotmail.com (H.A.F.); 2 Center for Materials Science, Zewail City of Science and Technology, October Gardens, Sixth of October 12578, Egypt; rraouf@zewailcity.edu.eg (R.R.H.); malkordi@zewailcity.edu.eg (M.H.A.); 3 Department of Microbiology and Immunology, Faculty of Pharmacy, Cairo University, Kasr El-Aini Street, Cairo 11562, Egypt; mohamed.abdelhalim@pharma.cu.edu.eg

**Keywords:** biofilms, bacterial adhesion, metal oxide nanoparticles (NPs), anti-adhesion, anti-biofilm, silicone, urinary catheter, grafting, poly-acrylic acid, gamma rays, ZnO nanoparticles, antibiofilm, *Pseudomonas aeruginosa*, gene expression, qRT-PCR

## Abstract

Nosocomial infections caused by microbial biofilm formation on biomaterial surfaces such as urinary catheters are complicated by antibiotic resistance, representing a common problem in hospitalized patients. Therefore, we aimed to modify silicone catheters to resist microbial adherence and biofilm formation by the tested microorganisms. This study used a simple direct method to graft poly-acrylic acid onto silicone rubber films using gamma irradiation to endow the silicone surface with hydrophilic carboxylic acid functional groups. This modification allowed the silicone to immobilize ZnO nanoparticles (ZnO NPs) as an anti-biofilm. The modified silicone films were characterized by FT-IR, SEM, and TGA. The anti-adherence ability of the modified silicone films was evidenced by the inhibition of biofilm formation by otherwise strong biofilm-producing Gram-positive, Gram-negative, and yeast clinical isolates. The modified ZnO NPs grafted silicone showed good cytocompatibility with the human epithelial cell line. Moreover, studying the molecular basis of the inhibitory effect of the modified silicone surface on biofilm-associated genes in a selected Pseudomonas aeruginosa isolate showed that anti-adherence activity might be due to the significant downregulation of the expression of lasR, lasI, and lecB genes by 2, 2, and 3.3-fold, respectively. In conclusion, the modified silicone catheters were low-cost, offering broad-spectrum anti-biofilm activity with possible future applications in hospital settings.

## 1. Introduction

Healthcare-associated infections (HCAIs) are the most common complications affecting hospitalized patients, with the most prominent being urinary tract infections [1]. The leading cause of these infections is the use of an indwelling urinary catheter, which is easily colonized by microorganisms existing on the patient’s skin or mucosa and that can transfer from infection sites in the body, resulting in the formation of biofilm [2]. Biofilms are complex communities of microorganisms that adhere to any biological or non-biological surface and are encased in self-produced extracellular polymeric substances (EPS) [3], which serve as the skeletal structure of biofilms and account for 90% of the total mass of biofilms. EPS is composed of proteins, polysaccharides, lipids, and extracellular deoxyribonucleic acid. Once formed, this polymeric matrix works as a shield to protect the cells within it from exposure to innate immune defenses. It prevents the antibiotics from penetrating due to biofilm matrix barrier characteristics such as negatively charged biofilm components and very viscous compact EPS structures [4]. In addition, biofilm inactivates antibiotics through enzymatic breakdown or adsorption.

Generally, the antibiotic resistance of bacteria forming biofilms is 1000 times greater than that of planktonic microorganisms, which makes treatment more difficult [5,6,7] and increases morbidity and mortality. Biofilm formation on urinary catheters exhibits local and systemic adverse effects [8]. Locally growing microorganisms result in a prolonged inflammatory state that harms both the host tissues and the structure and performance of the medical device itself. Microorganisms can also move from established biofilms and colonize other body parts, causing widespread infections other than at the main local site of infection [9]. These infections might need surgical debridement and antimicrobial therapy, which have some shortcomings [10]. The surgical debridement of biofilm is very complicated and painful for the infected patient. Worse, it might result in infection recurrence owing to bacteria remaining after surgery, as biofilm-forming bacteria are firmly adherent to the surrounding tissue [10]. Antibiotic therapy is another common treatment strategy; unfortunately, the therapeutic effect of antibiotics is compromised due to the emergence of bacterial resistance. The problem of biofilm formation on urinary catheters cannot be managed without removing the infected catheter with biofilm [7], which is practically complicated, as biofilms are not visible via the naked eye and are not economically feasible. Hence, preventing biofilm formation is a more effective treatment strategy for biofilm infectious diseases.

The most straightforward approach is to apply antibiotics to the catheter surface; however, this isn’t particularly efficient because of the development of antibiotic resistance. As a result, several studies have focused on researching novel antimicrobial drugs. Biomedical researchers started to reuse inorganic metal nanoparticles as antibiotic alternatives [11] due to their ability to target multiple sites in microorganisms, so they can fight against microbial resistance, and their nano-size can enhance biofilm penetration, making them superior to conventional antibiotics [12]. Metals such as silver, gold, zinc, and metal oxide nanoparticles have been shown to have strong antimicrobial activity against a broad range of pathogenic microorganisms [13]. 

Among several metal oxides, ZnO NPs is one of the most vital antimicrobial reagents that have received significant attention recently because of their remarkable features, such as a high surface-to-volume ratio, low cost, long-term environmental stability, and human cell biocompatibility [14,15,16]. ZnO NPs exhibit broad-spectrum antimicrobial activities on different pathogenic Gram-positive, Gram-negative and yeast microbial strains [16,17,18,19,20,21,22,23,24,25]. Recent lines of evidence suggest that the production of reactive oxygen species (ROS) in bacteria results in bacterial death [26]. Moreover, zinc ions from the partial dissolution of ZnO NPs change the membrane permeability, leading to the leakage of nucleic acid from the bacterial cells, thereby identifying it as the most probable mechanism of ZnO NPs’ anti-biofilm activity [27,28].

Silicone is the most commonly used material in urinary catheters, especially for people with latex allergies. Silicone has been used in a wide range of biomedical applications because of its good mechanical properties, chemical stability, and biocompatibility [29,30]. Silicone is a conventional base polymer with no functional groups on its surfaces, with a chemical structure based on chains of alternate silicon and oxygen atoms. This inertness limits its capability to uptake ZnO NPs via soaking or impregnation. It should be modified to include reactive groups for antimicrobial agent immobilization [31]. As a result, a series of studies were initiated in an attempt to impregnate the silicone surface with anti-biofilm properties by grafting hydrophilic, synthetic polymers that can resist microbial attachment [32] and with reactive functional groups to immobilize antimicrobial ZnO NPs [33].

Gamma radiation-induced graft copolymerization is a simple and valuable technique for medical device modification. It is used to graft or functionalize polymers on the surface of polymeric biomaterials without altering their mechanical properties [34] and to achieve drug and enzyme loading/release [35,36]. It is one of the most versatile means for the molecular design of polymer surfaces to enhance their physical and chemical properties for specific applications [37]. The irradiation activates the silicone polymer substrate by creating free radicals on its surface. The free radicals of the silicone polymer backbone initiate polymerization reactions of the monomer on its surface. After the initiation, the propagation of monomer chains continues [38]. 

Acrylic acid (AAc) is one of the most popular monomers that is surface polymerized (grafted) onto different polymeric matrices by this grafting technique. AAc has a very high degree of reactivity due to the existence of a carbon-carbon double bond [39], which allows it to react with free radicals present on the base polymer rapidly and to bind covalently to generate poly-acrylic acid (PAAc) grafted on silicone base polymers [40]. PAAc is a highly biocompatible polymer that acts as a spacer to bind antimicrobial ZnO NPs to silicone surfaces. 

The pH-sensitive response of PAAc ascribed to carboxylic groups (COOH) allows for further chemical reactions to acquire or generate new functional groups [41]. In this context, the functionalization of urinary catheters with PAAc provides numerous binding points for the ionic interaction with Zn^++^ ions, which, once bound, can be subsequently reduced to form nanoparticles [9]. Therefore, in this study, we aimed to combine the advantage of ZnO NPs’ antimicrobial activity and gamma radiation-induced grafting to produce a modified urinary foley catheter that can prevent biofilm formation and HCAIs. Our study offered a quick and inexpensive one-step in situ inter-matrix synthesis method to immobilize ZnO NPs utilizing the carboxylic acid functional groups created on the silicone surface. We found that introducing ZnO NPs effectively lowered the risk of microorganism colonization by offering a broad-spectrum antimicrobial activity with low-cost modifying technologies and making them safe to use with antibiofilm properties.

## 2. Materials and Methods

### 2.1. Synthesis of Zinc Oxide Nanoparticles

Zinc oxide nanoparticles (ZnO NPs) were synthesized using a solvothermal method [42]. Briefly, 0.81 g (3.73 mmol) of zinc acetate di-hydrate was dissolved in 40 mL of absolute ethanol in a 100 mL Schott bottle and heated at 55 °C under constant vigorous stirring using a hot plate stirrer. Next, 0.29 g (7.46 mmol) of sodium hydroxide was dissolved separately in 320 µL of bi-distilled water and 25 mL of absolute ethanol in a 100 mL Schott bottle under the same conditions of zinc acetate di-hydrate. After both solids had dissolved, the sodium hydroxide solution was added dropwise to the zinc acetate solution slowly over 2.25 h to form a white precipitate. The suspension was then cooled down to room temperature. The synthesized white ZnO NPs were collected by centrifugation once at 2000 rpm for 5 min, discarding the supernatant, and washing thoroughly with 5 mL absolute ethanol. This step was repeated twice to remove any unreacted precursors. The obtained product was then dried at 60 °C and ground to form a powder, after which it was stored at room temperature. All chemicals used in the preparation of ZnO NPs were of HPLC grade and were manufactured by Fisher Scientific (Waltham, MA, USA).

### 2.2. Characterization of ZnO NPs

#### 2.2.1. X-ray Diffraction Analysis

We used different techniques to characterize the synthesized ZnO NPs. The crystal structure and primary crystal size were characterized using an XRD-6000 powder X-ray diffractometer (Shimadzu scientific instruments, Kyoto, Japan) at the National Center for Radiation Research and Technology (NCRRT)—Egyptian Atomic Energy Authority, Cairo, Egypt. X-ray tube target (copper), voltage (40 kV), current (30 mA), and scanning drive axis (Theta-2Theta) with a continuous scan mode at a scan speed of 8 (deg./min) and the scan range from 4–90 (deg.) were the used parameters. The particle size (D) of the sample was calculated by Scherrer’s relationship [43]:

(1) $$D=\frac{0.94\lambda }{\beta cos\theta }$$

where λ is the X-ray wavelength, β is the broadening of the diffraction line measured at half of its maximum intensity in radians, and θ is the Braggs diffraction angle.

#### 2.2.2. Transmission Electron Microscopy

The size and morphology of the ZnO NPs were investigated using a JEOL JEM-100CX Transmission electron microscope (TEM) (Tokyo, Japan) operated at 200 kV. TEM studies were prepared by drop casting ZnO NPs dispersed in ethanoic solution after sonication for 60 min onto a carbon-coated TEM grid. The film on the TEM grid was allowed to dry, and the excess solution was removed using blotting paper.

### 2.3. Preparation of ZnO NPs Suspension for Antimicrobial Activity

ZnO NPs suspension was aseptically prepared by weighing a certain amount of dry ZnO NPs powder and adding it to liquid media (water or microbial broth) to achieve the specified concentration. The ZnO NPs suspension was dispersed using a full-power ultrasonic bath sonicator (FB15051, Fisher Scientific, Waltham, MA, USA) for 1 h with vigorous vortexing every 5 min for good dispersion of the nanoparticles in solution before performing the experiment [21]. The antimicrobial activity tests of the prepared ZnO NPs were carried out using different NPs concentrations against specific clinical isolates using minimum inhibitory concentration and anti-biofilm testing, as detailed below.

### 2.4. Isolation of Microbial Pathogens, Maintenace of Isolates and Standard Microbial Strains 

Different microbes were isolated from 36 discarded silicone urinary catheters previously used by intensive care unit (ICU) patients in Cairo, Egypt. Small segments from the catheters were placed in a phosphate-buffered saline (PBS) with a pH of 7.2 and then sent to the medical microbiology laboratory, where segments were cut longitudinally, vortexed, and bath sonicated for 5 min to detach the microbial biofilm on the catheter segments [44]. Different Gram-negative bacilli, Gram-positive cocci, and yeast were isolated using standard microbiological media, including nutrient agar, differential selective MacConkey Agar No. 3 media and Sabouraud Dextrose agar for yeast (Oxoid, Hampshire, UK). Pure isolates were obtained by culturing on different agar media using the streak plate method to select Gram-negative bacteria and differentiate them based on lactose fermentation into lactose fermenters and non-fermenters. Gram staining was performed for isolates to examine the Gram reaction and morphology for Gram-positive cocci and Gram-negative bacilli. 

Standard bacterial strains were used as controls, including *Pseudomonas aeruginosa* (PAO1), *Klebsiella pneumoniae* (2S11122), *Escherichia coli* (K12MG1655), *Staphylococcus aureus* (MRSA—N315), and *Staphylococcus aureus* (MSSA—RN6319). Standard strains were cultivated in nutrient broth or MacConkey Agar for identification tests or in Mueller-Hinton (MH) broth for MIC experiments. Stock cultures for isolates and standard strains were preserved in Luria broth (Oxoid, Hampshire, UK) containing 15% (*v*/*v*) glycerol at −80 °C. Antimicrobial susceptibility testing and biochemical and molecular identification of the selected clinical isolates was performed, as detailed in Section 2.6. 

### 2.5. Quantitative Biofilm Formation by Clinical Isolates

From 36 silicone urinary catheters, 74 pure cultures were isolated as detailed in Section 2.4 based on Gram staining, morphology, and the lactose fermentation for Gram-negative bacilli. Next, to detect the ability and classify the degree of biofilm formation, we used quantitative a crystal violet microtiter plate (MTP) assay [45,46]. Briefly, fresh overnight colonies of each isolate were inoculated in tryptic soy broth (TSB) (Oxoid, Hampshire, UK) with 1% glucose, incubated at 37 °C for 24 h, adjusted to 0.5 McFarland and diluted to 10^6^ colony forming units (CFU)/mL. Each well of sterile 96 well flat bottom polystyrene tissue culture treated plates (Sigma-Aldrich, Costar, St. Louis, MO, USA) was inoculated with 200 μL of prepared culture. Wells containing media alone represented negative controls. The plates were incubated at 37 °C for 24 h after incubation, and the contents of the wells were removed and washed three times with 200 μL PBS (pH 7.2). The biofilm was fixed by drying for 1 h at 60 °C, then stained with crystal violet solution (0.1% *w*/*v*), and left at room temperature for 15 min. Excess dye was removed by washing, and the plate was then left to dry. Biofilm-bound dye was solubilized with 95% ethanol, and the optical density (OD) was measured at 630 nm using a microplate reader (HumaReader HS, Wiesbaden, Germany). The average OD values were calculated for all tested isolates and negative controls, and the optical density cutoff value (ODc) was defined as three standard deviations (SD) above the mean OD of the negative control. The interpretation of biofilm production, either strong, moderate, weak, or non-biofilm producing, was based on the previously established criteria of Stepanović et al., 2007 [46], where the microbial isolate was considered non-biofilm producing (0) at OD ≤ ODc, weak biofilm producing (+ or 1) at ODc < OD ≤ 2 × ODc, moderate biofilm producing (++ or 2) at 2 × ODc < OD ≤ 4 × ODc, and strong biofilm producing (+++ or 3) at 4 × ODc < OD [46].

### 2.6. Biochemical and Molecular Identification and Antimicrobial Susceptibility Testing of Selected Strong-Biofilm Forming Clinical Isolates

Strong biofilm-producing microbial isolates (10 from 74 tested isolates) were identified to the species level by the use of the automated biochemical assay method using VITEK 2 Compact ID cards (BioMerieux, Marcy-l’Étoile, France). Next, the antimicrobial susceptibility of the ten-strong biofilm-forming isolates towards different antibiotic classes was also undertaken with VITEK 2 Compact AST cards (BioMerieux, Marcy-l’Étoile, France) according to the manufacturer’s instructions [47]. The Clinical and Laboratory Standards Institute (CLSI) performance standards for antimicrobial susceptibility testing (M100) breakpoints were used to categorize the results into three categories: resistant (R), intermediate (I), and Sensitive (S) [48]. Further classification of the isolates was done by dividing them into susceptible (S), multidrug-resistant (MDR), and extensively drug-resistant (XDR) according to the interim standard definitions for acquired resistance, as recommended by [49]. 

Since one of the strong biofilm-forming isolates was identified as *Candida tropicalis*, we additionally performed antifungal susceptibility testing by using the disk diffusion method where Nystatin (100 IU) antifungal discs (Oxoid, Hampshire, UK) were applied on Müller-Hinton agar (MHA) (Oxoid, Hampshire, UK) as recommended by [50]. The interpretation of nystatin antifungal susceptibility (susceptible S, susceptible dose-dependent [SDD], and resistant R) was made according to the Clinical Laboratory Standard Institute (CLSI) M44A document.

Six different microbial isolates from the ten-strong biofilm-producing isolates were selected for further study and were molecularly identified using 16S rRNA gene sequencing. Briefly, the DNA extraction was done according to the manufacturer’s instructions using a Quick-DNA Fungal/Bacterial Miniprep Kit (Zymo Research, Irvine, CA, USA). The genomic DNA quality was checked using agarose gel electrophoresis, where a thick high molecular band appeared above 10 Kb without smearing. The amplification of the *16S rRNA* gene was conducted using universal forward(8F) and reverse primers (1492 R) at 57–59 °C with a product size of 1500 bp [51]. PCR amplification was undertaken using Cosmo PCR RED Master Mix (W1020300x-Willowfort, Birmingham, UK) according to the manufacturer’s instructions. The quality of the PCR product was checked by gel electrophoresis, where a band at 1500b without any unspecific product was obtained. The PCR product was then purified using a DNA Clean & Concentrator Kit (DCC-Zymo Research, Irvine, CA, USA) and sequenced using the SANGER technique. Sequences were trimmed to remove the noise, then aligned using the BLAST server on the NCBI database to check for the best matching sequence using the (nr/nt) database.

### 2.7. Antimicrobial and Anti-Biofilm Effect of ZnO NPs 

#### 2.7.1. Determination of Minimum Inhibitory Concentration (MIC)

The MIC of ZnO NPs towards the selected clinical isolates was investigated using the agar dilution method according to CLSI M07 [52]. Briefly, 10X ZnO NPs suspensions were serially diluted by two-fold dilutions to obtain concentrations of 160 to 0.625 mg/mL, then one part of each of these ZnO NPs suspensions was added to nine parts of molten MH agar (MHA). The final ZnO NPs agar concentrations ranged from 16 to 0.0625 mg/mL. The prepared ZnO NPs-MHA plates were left to cool down.

Microbial suspensions were prepared by the colony suspension method and adjusted to 10^6^ CFU/mL. Microbial suspensions (10 µL) were spotted (inoculum of 10^4^ CFU/spot) on prepared ZnO NPs-MHA plates. The inoculum spots were allowed to dry before inverting the plates to be incubated at 37 °C aerobically for 24 h. The agar plate without nanoparticles was considered a positive growth control. The MIC was recorded as the lowest concentration of ZnO NPs that completely inhibited growth according to CLSI M07 [52].

#### 2.7.2. Effect of ZnO NPs on Growth and Biofilm Formation at Sub-MIC

The activity of ZnO NPs against the biofilm formation of the selected clinical isolates was investigated using the same technique detailed in Section 2.5 with the modification of supplementing the medium with different concentrations of ZnO NPs (1 to 0.0625 mg/mL) [53,54]. Negative color controls of different concentrations of ZnO NPs without microbes and positive growth controls of microbes without ZnO NPs were performed for ODc value calculations. Each plate was incubated at 37 °C for 24 h. After incubation, 10 µL from each well was spotted on a fresh nutrient agar plate to examine the survival (viability) of tested microbes; the plates were then treated as described in Section 2.5. The average OD values were calculated and compared to the cutoff value (ODc) according to [46], as previously detailed in Section 2.5.

### 2.8. Gamma Radiation-Induced Graft Copolymerization of AAc onto Silicone Rubber Polymer for Functionalization

**Scheme 1:**
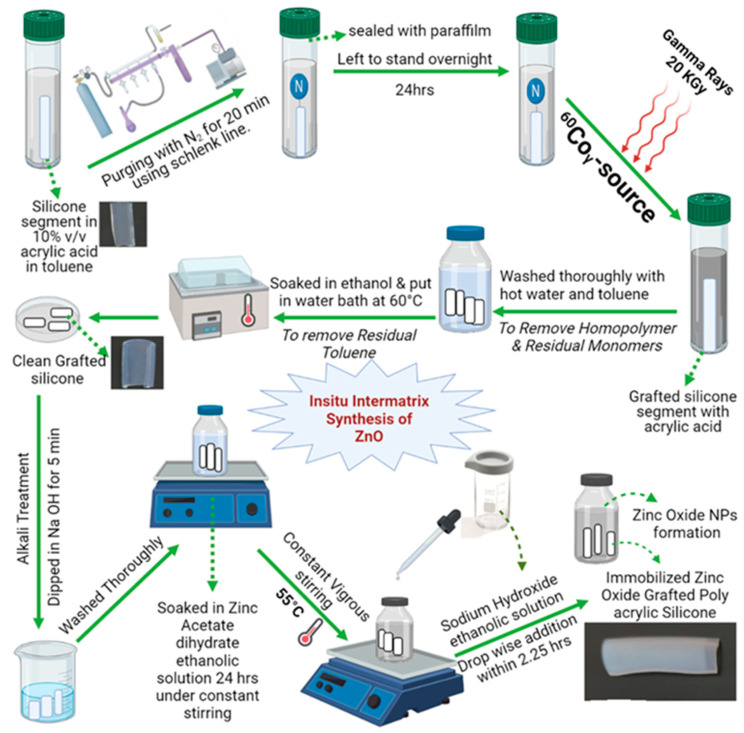
Overview of the grafting copolymerization of acrylic acid (AAc) (10% *v*/*v*) onto the silicone segment using ^60^Co gamma irradiation (20 kGy) and the in situ inter-matrix synthesis of ZnO nanoparticles (NPs) on poly-acrylic acid grafted silicone (SR-g-AAc) (Figure created with BioRender.com).

Silicone catheter segments were prepared from a commercially available Foley Catheter (Well Lead, Medical Co., Guangzhou, China) and functionalized by an acrylic acid monomer using gamma radiation-induced direct grafting copolymerization while studying the effect of monomer concentration and the dose of radiation on the grafting yield [55,56]. Briefly, untreated pristine silicone segments (SR) were washed with alcohol and dried at 80 °C until constant weight (W_0_). Segments were placed in glass vials containing either 5, 10, or 20% *v*/*v* monomer solutions of acrylic acid (98% stabilized with HQ) (ADVENT CHEMBIO PVT, Mumbai, India) in toluene as a solvent (HPLC grade, Waltham, Massachusetts, USA), exposing them to a constant radiation dose of 20 kGy for the monitoring of the monomer concentration effect. In addition, for monitoring the effect of the radiation dose, segments were placed in glass vials containing a constant monomer concentration of 10% *v*/*v* while exposed to different doses of radiation: 10, 15, or 20 kGy. The vials were degassed by purging the solution with N_2_ using the Schlenk line for 20 min. Then, without further exposure to air, the vials were closed and sealed. Irradiation was performed at the NCRRT, Egyptian Atomic Energy Authority, Cairo, Egypt using a ^60^Co Gamma Chamber 4000-A-India at a dose rate of 1 kGy/h. The obtained poly-acrylic acid grafted silicone segments (SR-g-AAc) were washed thoroughly with different solvents to remove residual monomers and the homo-polymer formed during the reaction and then dried to a constant weight (W_g_), as shown in Scheme 1. The effect of monomer concentration and radiation dose on the grafting yield was evaluated by calculating the grafting percent. The grafting yield percent was calculated according to Equation (2) based on Vázquez-González et al. [34]:

(2) $$\mathrm{GY}\;\%=\left[\frac{\;(W_{g}-W_{0})\;}{W_{0}}\right]\times \;100$$

where W_0_ represents the weight of the initial segment (SR), and W_g_ represents the weight of grafted segments (SR-g-AAc).

### 2.9. Immobilization of ZnO NPs on SR-g-AAc

ZnO NPs were immobilized on SR-g-AAc using the wet in situ inter-matrix synthesis method as described by d’Água et al. [57] with slight modifications. Briefly, SR-g-AAc segments were first dipped in 0.1 M sodium hydroxide ethanolic solution (alkali treatment) for 5 min to ionize the COOH groups in the grafted PAAc chains for activating the ionic interactions with oppositely charged zinc ions. The activated segments were then washed thoroughly to remove excess sodium hydroxide and were subsequently immersed in 0.1 M zinc acetate dihydrate solution under constant magnetic stirring at room temperature for 24 h to allow the ionic interactions of the cationic zinc ions with the activated carboxylic acid groups. Afterward, under the same constant vigorous stirring, the temperature was raised to 55 °C, and 0.2 M NaOH ethanolic solution was added dropwise over 2.25 h at a rate of 1 mL/5 min to produce and immobilize ZnO NPs on SR-g-AAc segments (Scheme 1). Modified ZnO NPs grafted silicone segments (SR-g-AAc-ZnO) were washed thoroughly with water and ethanol and dried. The amount of ZnO immobilized was thermogravimetrically quantified.

### 2.10. Characterization of the Modified Silicone Polymeric Material

The grafting and immobilization modifications were investigated by multiple techniques, which were performed according to the manufacturer’s instructions.

#### 2.10.1. Fourier Transform Infrared Spectroscopy

Infrared Spectroscopy was performed on SR, SR-g-AAc, and SR-g-AAc-ZnO using a Nicolet iS10 FTIR Spectrometer (Thermo Fisher Scientific, Waltham, MA, USA) at a scanning range of 4000 to 650 cm^−1^ wavenumber, in order to investigate the change in silicone structure and confirm the modification.

#### 2.10.2. Scanning Electron Microscope and Energy Dispersive Spectroscopy Analysis

The microstructure surfaces of SR and SR-g-AAc were analyzed by a Scanning electron microscope (SEM) (ZEISS Sigma 300 VP, Oberkochen, Germany), coupled with energy dispersive X-ray spectrometry (EDX), normally operating at 15 kV. 

#### 2.10.3. Thermogravimetric Analysis

A thermogravimetric analysis (TGA) was performed on SR, SR-g-AAc, and SR-g-AAc-ZnO. The thermal decomposition was determined under a nitrogen atmosphere between 30 °C and 700 °C at a heating rate of 10 °C/min using TGA Q50 (TA Instruments, New Castle, DE, USA). 

### 2.11. Cytocompatibility Assay of SR-g-AAc-ZnO (Extract Dilution Cell Culture Assay)

The cytocompatibility of SR-g-AAc-ZnO was employed to evaluate the potential toxicity of leachable substances if present in their extracts and compared with SR [34]. The SR-g-AAc-ZnO and SR segments were placed separately in 1 mL of Dulbecco’s Modified Eagle’s Medium (DMEM) (Lonza, Basel, Switzerland) supplemented with 100 mg/mL of streptomycin, 100 units/mL of penicillin (Lonza, Basel, Switzerland), and 10% of heat-inactivated fetal bovine serum (Gibco, Thermo Fisher Scientific, Waltham, MA, USA) (hereafter referred to as complete cell culture medium) and incubated for 24 h at 37 °C for extraction. The extract of the modified silicone was then diluted both two- and four-fold. The cytocompatibility of the modified silicone was assessed by testing the viability of normal human oral epithelial cells (OEC) in the presence of different modified silicone extracts compared with non-treated silicone extract and negative control (cells without extract).

A sulforhod-amine B (SRB) assay was used to test the cell viability of OECs from Nawah Scientific Inc. (Mokattam, Cairo, Egypt) as described in [58,59]. Cells were cultured in the aforementioned complete cell culture medium and incubated at 37 °C under a humidified atmosphere with 5% CO_2_. When the cells reached confluence, aliquots of 100 µL of 5 × 10^4^ cells/mL were seeded to 96 well plates and incubated at 37 °C in a 5% CO_2_ atmosphere for 24 h to facilitate cell adherence to the bottom of the wells. Cells were treated with another aliquot of 100 µL from each extracted sample. Wells with no added extract acted as a negative control. After 72 h of extract exposure, cells were fixed by replacing the media with 150 µL of 10% trichloroacetic acid (TCA) (Merk & Co., Darmstadt, Germany) and incubated at 4 °C for 1 h. The solution with TCA was removed, and the cells were washed five times with distilled water. Aliquots of 70 µL SRB solution (0.4% *w*/*v*) (Sigma Aldrich, Darmstadt, Germany) were added and incubated in the dark at room temperature for 10 min. The plates were washed three times with 1% acetic acid and allowed to air-dry overnight. Next, 150 µL of Tris-HCl (10 mM) was added to dissolve the protein-bound SRB stain for measuring absorbance (OD) at 540 nm using a BMG LABTECH^®^-FLUOstar Omega microplate reader (Ortenberg, Germany). Tests were done in triplicate. Negative controls were also prepared by adding fresh culture medium to cells and treating them similarly. The cell viability was calculated according to Equation (3) based on Orellana and Kasinski [60]:

(3) $$Cell\;viability\left(\%\right)=\frac{Absorbance\;Sample}{Absorbance\;Negative\;Control}\times 100$$

### 2.12. Anti-Adherence Activity of the Irradiated and Non-Irradiated SR-g-AAc-ZnO against Biofilm Producing Isolates

Some SR-g-AAc-ZnO segments were gamma irradiated with an absorbed 25 kGy (sterilization dose) at 1 kGy/h of intensity. The anti-adherence activity of SR-g-AAc-ZnO (5 mm × 5 mm) was studied as described in [61,62]. Briefly, the selected clinical isolates were plated and incubated on nutrient agar overnight, then suspended in TSB diluted in PBS (Lonza, Basel, Switzerland) to reach an approximate cell density of 10^6^ CFU/mL in 1% TSB. For the adhesion assay, segments from SR and non-irradiated SR-g-AAc-ZnO were rinsed with 70% ethanol for 20 min for sterilization purposes and were left in the sterile Petri dishes for 30 min until they reached complete dryness. All segments of untreated SR, irradiated, and non-irradiated SR-g-AAc-ZnO were then transferred into a 24-well plate and treated with the prepared 1 mL of the microbial suspensions and incubated at 37 °C for 72 h. Each segment was removed from the suspensions aseptically at a specified time, and the non-adherent cells were removed by washing with 0.9% normal saline. They were then placed in a tube containing 1 mL PBS to be sonicated and vortexed to detach the adhered biofilm microbial cells, and later plated on a nutrient agar by Miles and Misra’s technique following 10-fold serial dilutions [63]. After overnight incubation at 37 °C, the CFUs on the plate were counted and converted to CFU/segment. All experiments were repeated as three independent biological replicas. 

#### Scanning Electron Microscope Analysis

Segments of SR and non-irradiated SR-g-AAc-ZnO for *Staphylococcus aureus* representing Gram-positive cocci, *Klebsiella pneumoniae* representing Gram-negative bacilli, and *Candida tropicalis* representing yeast isolates were collected. The segments were then processed for imaging using an SEM (ZEISS EVO 15, Oberkochen, Germany) operated at 25 kV, according to the procedure of Storti et al. [64]. 

### 2.13. Effect of SR-g-AAc-ZnO Surface on Adhered Pseudomonas aeruginosa Isolate Differential Gene Expression of Biofilm-Associated Genes

Briefly, the *P. aeruginosa* identified isolate was cultured in TSB, and growth was adjusted to 10^8^ CFU/mL and incubated at 37 °C for three days in contact with either SR or non-irradiated SR-g-AAc-ZnO segments. After three days of incubation, silicone segments were removed and suspended in PBS, vortexed, and sonicated for five minutes to detach the adhered cells from the segments. The adhered cells were resuspended in PBS and pelleted by centrifugation at 13,000× *g* for 5 min at 4 °C. After harvesting the adhered cells, the RNA was extracted using a RNeasy mini kit (Qiagen, Hilden, Germany). Genomic DNA was removed by on-column digestion for 25 min using a RNase-free DNase set (Qiagen, Hilden, Germany) according to the manufacturer’s protocol. RNA concentrations were determined spectrophotometrically at an absorbance of A_260_ using a nanophotometer (Implen P330, Munich, Germany). The absorbance ratio at 260 nm to 230 nm and 280 nm was used to assess the purity of the extracted RNA. The quality of RNA was further evaluated via electrophoresis on 1.8% agarose with sharp bands corresponding to 16S and 23S rRNA. 

Purified RNA was used for cDNA synthesis using the High-Capacity cDNA Reverse Transcription Kit (Thermo Scientific, Waltham, MA, USA) according to the manufacturer’s instructions. A starting RNA concentration of 10 ng/µL was chosen for all samples. cDNA concentrations were determined by a nanophotometer, and cDNA samples were stored at −20 °C until use. 

A real-time PCR was carried out using a Quantifast SYBR green real-time PCR kit (Qiagen, Hilden, Germany) following the manufacturer’s protocol. The cycling conditions were as follows: PCR initial heat activation at 95 °C for 5 min followed by 40 cycles of 2-steps; denaturation at 95 °C for 10 s, then combined annealing/extension at 60 °C for 30 s using a Qiagen Rotor-Gene Q-5 Plex real-time thermal cycler system (Qiagen, Hilden, Germany). A melting curve analysis was performed with a ramp from 55 °C to 99 °C, raising the temperature by 1 degree and waiting 5 s for each step. A single sharp peak in the melting curve analysis demonstrated that the primers were specific to the target genes. Each target gene’s threshold cycle (Ct) was first normalized to the selected housekeeping gene 30S ribosomal protein S12 (*rpsl*). The differential gene expression was then expressed as the fold change relative to cells that adhered to non-treated silicone. The fold change was determined using the 2^−∆∆Ct^ method [65]. Primers used in this study are listed in Table 1 and were manufactured by Macrogen, Inc. (Seoul, South Korea). Assays were performed in technical duplicates, and the results represent the means of two independent biological replicas.

### 2.14. Statistical Analysis

A statistical analysis was performed using Graph Pad Prism 8 software (GraphPad Software, San Diego, CA, USA). The experimental results were expressed as mean ± SD for technical replicas from at least two different biological replicas. The results were analyzed using a one-way ANOVA and a Student’s *t*-test. Generally, statistical significance was defined as a * *p* < 0.05, ** *p* < 0.01, *** *p* < 0.001, **** *p* < 0.0001. For testing the significance of the anti-biofilm activity of ZnO NPs at different concentrations compared to without ZnO, a one-way ANOVA followed by a post hoc Dunnett’s test was employed. This was also used to test the significance of the anti-adherence activity of modified ZnO grafted silicone compared to untreated silicone. A Student’s *t*-test was performed to test the significant fold change in gene expression analysis using real-time PCR. 

## 3. Results

### 3.1. Characterization of the Synthesized ZnO NPs

#### 3.1.1. X-ray Diffraction Analysis

The synthesized ZnO NPs were characterized using Powder X-ray diffraction (PXRD). The PXRD showed peaks at 2θ = 31.6°, 34.3°, 36.1°, 47.4°, 56.4°, 62.7°, and 67.9°, corresponding to the (100), (002), (101), (102), (110), (103), and (112) planes of ZnO, respectively, as shown in Figure S1. The results matched well with the JCPDS file #79-0207 of the standard ZnO, which can be indexed as corresponding to the reflection of the hexagonal crystal structure. No characteristic peaks of impurity phases were observed. The crystalline particle size was calculated using Debye Scherrer’s equation, which was found to be 10.3 nm based on the peak value of 36.1°, the peak of highest intensity from the full-width at half-maximum (FWHM). 

#### 3.1.2. Transmission Electron Microscopy

TEM is a reliable technology for NPs characterization. TEM was used to determine the actual particle size and shape pattern with high precision. TEM micrographs of the produced ZnO NPs revealed a uniform shape that that appeared hexagonal or spherical with a consistent size distribution and an average particle diameter of 14 nm, as shown in Figure S2.

### 3.2. Microbial Isolation, Biofilm Detection, Identification, and Sensitivity of Strong Biofilm Clinical Isolates

A total of 74 microbial isolates were recovered from 36 silicone catheter segments. These were categorized based on conventional microbiological methods using culturing and microscopical characterization into 46 (62.1%) mainly Gram-negative bacilli as expected with urinary catheters, 10 (13.5%) Gram-positive cocci, and 18 (24.3%) yeast (Table S1). The biofilm production of these isolates revealed that 64 (86.4%) of them could produce biofilm (Table S2). Out of these 64 biofilm-producing isolates, 10 isolates (15.6%) were strong biofilm producing based on Stepanović et al.’s (2007) [46] categorization of biofilm production, and these ten isolates were selected for further characterization.

The selected ten-strong biofilm-producing isolates were further identified by a VITEK 2 compact system, showing that Gram-negative non-lactose fermenter bacilli were *Pseudomonas aeruginosa* (three isolates) and *Alcaligenes faecalis* (one isolate) (Table S3). The Gram-negative lactose fermenter bacilli were *Klebsiella pneumoniae* (three isolates) and *Escherichia coli* (one isolate) (Table S3). As for the Gram-positive cocci, one isolate was identified as *Staphylococcus aureus* and one yeast isolate was identified as *Candida tropicalis* (Table S3).

Next, the antimicrobial susceptibility test (AST) was performed using a nystatin antifungal sensitivity test for the identified *C. tropicalis* and a VITEK 2 Compact system for the nine-strong biofilm-producing bacterial isolates (Tables S4 and S5). The nystatin disc showed no inhibition zone; thus, the tested *C. tropicalis* is resistant to nystatin according to CLSI breakpoints. Three of the strong biofilm-producing bacterial isolates showed multiple antibiotic resistance (Table S6). Next, we further identified these isolates along with *A. fecalis*, *S. aureus*, and yeast *C. tropicalis* by molecular identification using *16S rRNA* gene sequencing, as shown in Table 2. Next, we tested the effect of ZnO NPs on growth inhibition and biofilm formation in the molecularly identified six selected isolates.

### 3.3. Effect of ZnO NPs on Growth and Biofilm formation by Selected Clinical Isolates

#### 3.3.1. Determination of Minimum Inhibitory Concentration of ZnO NPs

The MIC of the synthesized ZnO NPs suspension was determined against the six strong biofilm microbial isolates compared to standard strains (Table 3). It was observed that ZnO NPs showed a good inhibitory effect, with MIC ranging from 0.25 to 2 mg/mL for most of the tested isolates and their standard strains. An exception was for *P. aeruginosa* (16 mg/mL), as it was an XDR isolate (Table S6), and *C. tropicalis* (8 mg/mL), which is resistant to nystatin (Table 3).

#### 3.3.2. Determination of the Effect of ZnO NPs on Biofilm Formation at Sub-MIC 

**Figure 1:**
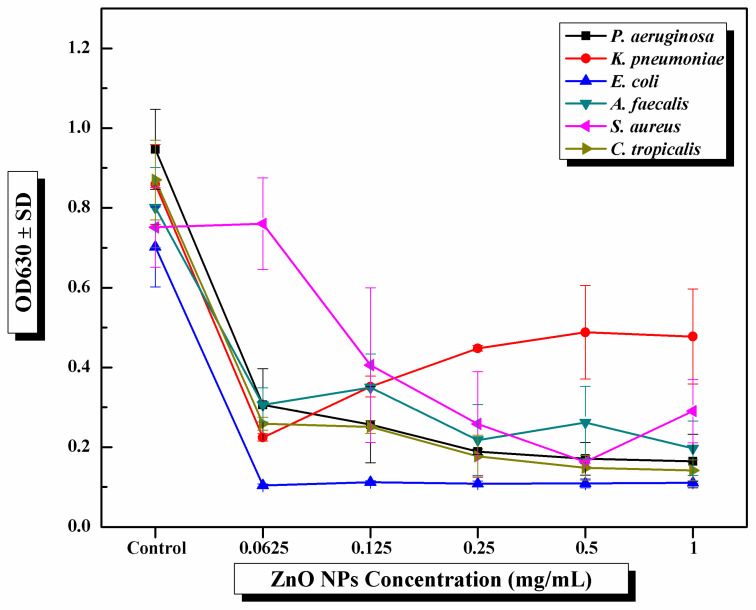
Effect of ZnO NPs at subMIC concentrations on the biofilm formation of the selected strong biofilm forming isolates. The biofilm formation was quantified by OD630 and compared to the optical density cutoff value (ODc). Results are displayed as the mean OD630 ± SD, which significantly decreased at ZnO NPs of 0.0625 mg/mL and at a ZnO NPs concentration of 0.125, 0.25, and 1 mg/mL using a one-way ANOVA followed by a post hoc Dunnett’s test.

ZnO NPs exhibited a strong anti-biofilm activity at different sub-MIC concentrations against the six tested strong biofilm-producing microbes (Figure 1 and Table S7). This anti-biofilm activity was observed by the significant decrease in the average ODs630 recorded after challenging each microbe with different ZnO NPs concentrations. Although the MIC of ZnO NPs was 16 mg/mL towards *P. aeruginosa*, using a sub-MIC of 0.0625 mg/mL of ZnO NPs showed significant anti-biofilm activity. We compared the average OD values with the optical density cutoff value (ODc) equal to 0.158, where ODc is defined as three standard deviations (SD) above the mean OD of the negative control, as previously detailed in Section 2.5. The strong biofilm-producing ability of *P. aeruginosa*, *A. faecalis*, and *C. tropicalis* became weak after exposure to sub-MIC of ZnO NPs. At the same time, the biofilm-producing ability was inhibited entirely in the case of *E. coli*. Regarding *K. pneumonia* and *S. aureus*, their strong ability to produce biofilm became moderate after exposure to the sub-MIC of ZnO NPs. We then aimed to modify the silicone catheter with the synthesized ZnO NPs and to test the ability of the modified ZnO NPs silicone to prevent bacterial adhesion and biofilm formation.

### 3.4. Optimization Factors Affecting Radiation-Induced Graft Copolymerization of AAc on Silicone Catheter Segments

**Figure 2:**
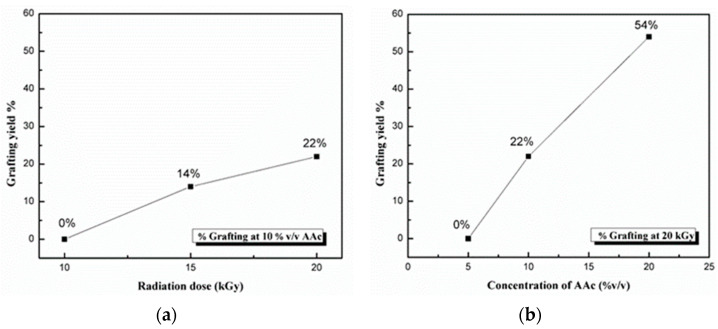
Factors affecting grafting yield percent. (**a**) Effect of different gamma irradiation doses (kGy) on the grafting yield%; (**b**) Effect of different AAc monomer concentrations (% *v*/*v*) on the grafting yield %.

It was observed that the degree of grafting was affected by the radiation dosage and the monomer concentration. As shown in Figure 2a, the grafting yield % increased as the radiation dose increased from 10 to 20 kGy, which may result from more breaking of covalent bonds in the silicone structure, so there was a more radical formation to graft more chains of PAAc. Regarding the effect of monomer concentration (Figure 2b), the degree of grafting increased with the increase in the monomer concentration from 5 to 20% *v*/*v,* as more AAc diffused to silicone polymer came in contact with the active sites of the irradiated silicone for polymerization. Although increasing the grafting yield of PAAc on silicone will provide more functionalized sites for the immobilization of ZnO NPs, a more whitish appearance and greater rigidity of silicone grafted with 54% was observed. Thus, silicone grafted with 22% PAAc was used for further study. 

### 3.5. Characterization of SR-g-AAc Silicone Catheter 

**Figure 3:**
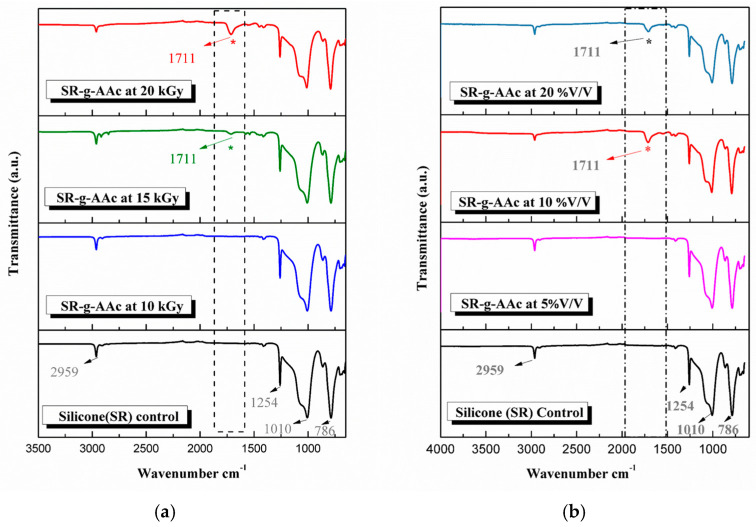
Fourier-transform infrared spectra (FTIR) of silicone segments before and after grafting with acrylic acid: (**a**) FTIR of pristine untreated silicone (SR) and silicone exposed to different gamma irradiation doses (10, 15, 20 kGy) at constant AAc monomer concentration (10% *v*/*v*). (**b**) FTIR of SR and silicone gamma irradiated at 20 kGy using different AAc monomer concentrations (5, 10, 20% *v*/*v*). * The appearance of an additional peak at 1711 cm^−1^ corresponded to the stretching vibration of the carbonyl group (C=O), indicating the successful grafting of PAAc onto the surface of silicone.

FTIR spectroscopy was employed to investigate the changes in the chemical structure of silicone because of the grafting process. An FTIR study was done on silicone after grafting using different radiation doses at 10% *v*/*v* monomer concentration, as shown in Figure 3a. Figure 3b shows the changes using different monomer concentrations at a 20 kGy radiation dose. The spectrum of pristine SR showed a band at 1010 cm^−1^ due to the stretching vibration (νstr) of the Si-O-C bond; 1254 cm^−1^ and 786 cm^−1^ correspond to Si-CH_3_ and 2959 cm^−1^ related to C-H groups in CH_3_. Upon increasing the radiation dose (15, 20 kGy) as in Figure 3a, and the monomer concentration (10, 20% *v*/*v*) as in Figure 3b, an additional peak appeared at 1711 cm^−1^. This can be ascribed to the νstr of the carbonyl group (C=O), which shows an absorption band between 1650 to 1800 cm^−1^, indicating the successful grafting of PAAc onto the surface of the silicone. In addition, SEM was performed to visualize the smooth surface of SR film, showing no observable discontinuities in its structure (Figure S3a,b). After grafting, SR-g-AAc (Figure S3c,d) showed a granular surface.

### 3.6. Characterization of SR-g-AAc-ZnO Silicone Catheter

#### 3.6.1. Fourier Transform Infrared (FTIR) Spectroscopy Analysis 

**Figure 4:**
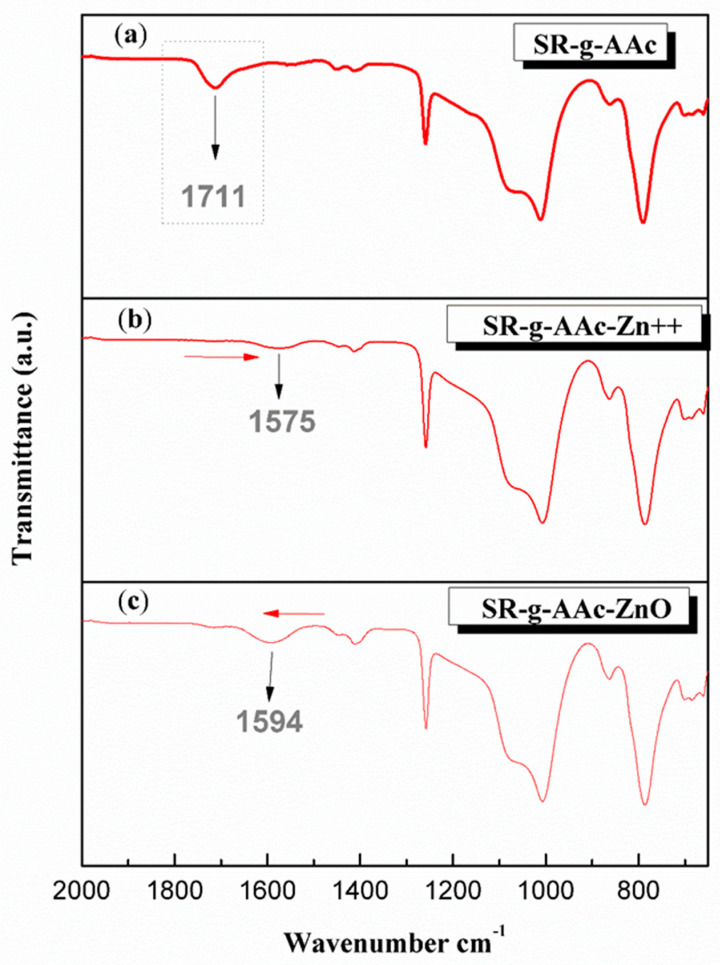
(**a**) FTIR of SR-g-AAc with the characteristic peak at 1711 cm^−1^ corresponding to the carbonyl of PAAc; (**b**) FTIR after soaking of SR-g-AAc in zinc acetate and shifting to a lower wavenumber of 1575 cm^−1^, (**c**) FTIR after the reduction of Zinc ions chelated to PAAc to ZnO NPs.

Poly-acrylic acid grafted silicone has been effectively used for the immobilization of ZnO NPs. The characteristic carbonyl peak at 1711 cm^−1^ (Figure 4a) of the carboxylic group (COOH) due to the grafting of poly-acrylic acid (Figure 4a) was shifted to a lower wavenumber of 1575 cm^−1^ after soaking in zinc acetate dihydrate (Figure 4b). This can be ascribed to the νstr of the carboxylate group that shows an absorption band at a lower wavenumber between 1550 to 1620 cm^−1^, indicating the successful polarization of COOH and ionic interaction with Zinc ions as shown in Figure 4b. After the reduction and formation of ZnO NPs, the carboxylic group becomes partially polarized, shifting the peak back to a slightly higher wavenumber at 1594 cm^−1^ (Figure 4c).

#### 3.6.2. Energy Dispersive X-ray Spectroscopy (EDX) Analysis 

**Figure 5:**
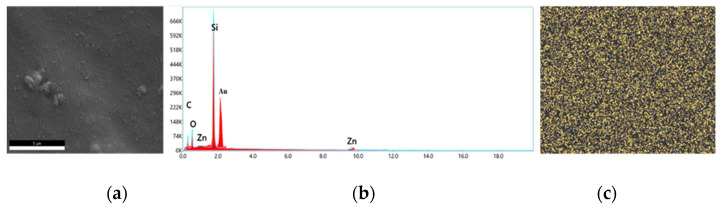
(**a**) SEM micrographs of modified ZnO NPs grafted silicone (SR-g-AAc-ZnO) operated at 15 kV; (**b**) Results of SEM- Energy Dispersive X-ray Spectroscopy (EDX) spectrum prove the successful modification of silicone by ZnO NPs, (**c**) SEM-EDX mapping at a magnification of 16.00 KX showing a homogenous coating of ZnO NPs (yellow dots on the figure) as proof of successful modification.

SEM-EDX results proved that the modified silicone was impurity-free and immobilized by ZnO NPs. Figure 5a shows an SEM microphotograph of the silicone surface with immobilized ZnO NPs. Figure 5b shows the EDX spectra of this surface, with the characteristic peaks for Si and C typically found in the pristine SR, the characteristic peak of Au due to the gold sputtering of the sample, and the appearance of peaks for both zinc and oxygen, confirming the successful modification and immobilization of ZnO NPs. The mapping (Figure 5c) showed a uniform homogenous coating over the modified silicone surface, with an equal distribution of ZnO NPs (yellow dots in the figure). 

### 3.7. Thermogravimetric Analysis

**Figure 6:**
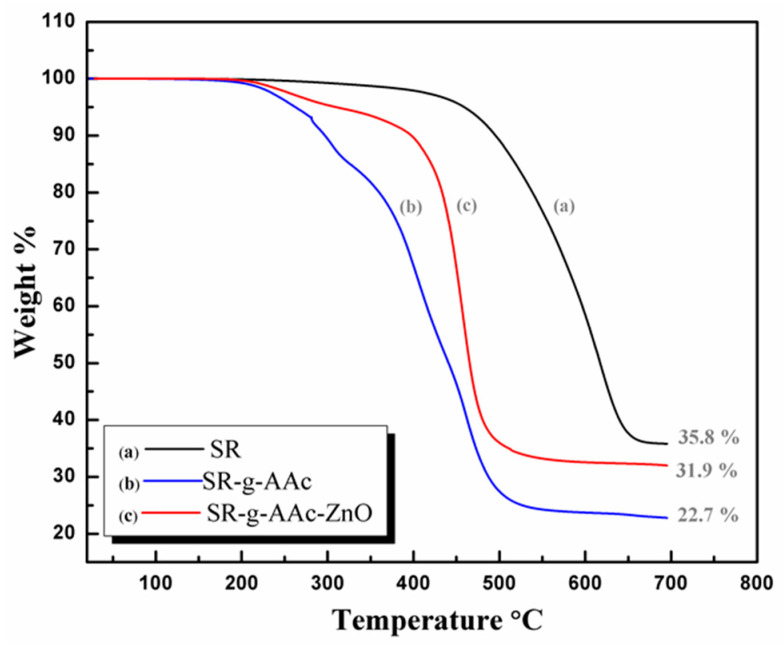
Thermogravimetric analysis of treated silicone with immobilized ZnO NPs. (a) Thermogravimetric analysis (TGA) of SR showing thermal stability below 400 °C; (b) Thermogram change after PAAc grafted on silicone (SR-g-AAc) showing additional two decomposition steps and proving the successful grafting of PAAc, (c) Thermogram of SR-g-AAc-ZnO showing better thermal stability after immobilization of ZnO NPs on grafted silicone.

The thermogravimetric curves show that the pristine SR is thermally stable below 400 °C and starts decomposition at 470 °C, as shown in Figure 6(a). After the grafting of silicone by PAAc (Figure 6(b)), three decomposition stages were observed. The first decomposition step occurred in the 200–280 °C range, which is related to the anhydrization of PAAc. The second decomposition step, from 370 to 470 °C, can be attributed to the degradation of the main chain of PAAc. Finally, the third decomposition starts at 470 °C due to the thermal decomposition of the silicone. According to these results, the grafting of silicone by AAc was successfully achieved. Regarding the thermogram after immobilization of the ZnO NPs on the SR-g-AAc (Figure 6(c)), a slowdown in the decomposition of the SR-g-AAc was observed, which can be attributed to the chelation of ZnO NPs to the PAAc molecules chain, giving the grafted silicone better thermal stability. Furthermore, it was observed that the final remaining weight percentage was 9.2% higher than that of the grafted silicone, corresponding to the percentage of immobilized ZnO NPs to the total weight of the modified silicone. These findings suggest the successful immobilization of ZnO NPs on the grafted silicone.

### 3.8. Cytocompatibility Assay for SR-g-AAc-ZnO

The cytocompatibility of SR-g-AAc-ZnO was tested, as the potential toxicity of any leachable substances from SR-g-AAc-ZnO was compared to the pristine SR polymer. The results showed good cytocompatibility with OEC relative to the negative control (Table 4) where cell viability values were above 98%.

### 3.9. Anti-Adherence Activity of the Irradiated and Non-Irradiated SR-g-AAc-ZnO against Biofilm Producing Isolates

SR-g-AAc-ZnO segments showed significant inhibition (*p* < 0.05) in the adherence of the six-strong biofilm-producing selected isolates compared to SR. The SR-g-AAc-ZnO, either non-irradiated or irradiated, completely inhibited the adherence of *P. aeruginosa*, *E. coli*, and *S. aureus,* with no growth detected (Table 5). At the same time, the adhesion of *K. pneumoniae* was reduced by 1 or 2 logs for the non-irradiated and irradiated SR-g-AAc-ZnO, respectively. The effect was less for *A. faecalis*, as less than 1 log reduction was observed with both modified silicones. However, the adherence of *C. tropicalis* was not affected. 

#### Scanning Electron Microscope Analysis

**Figure 7:**
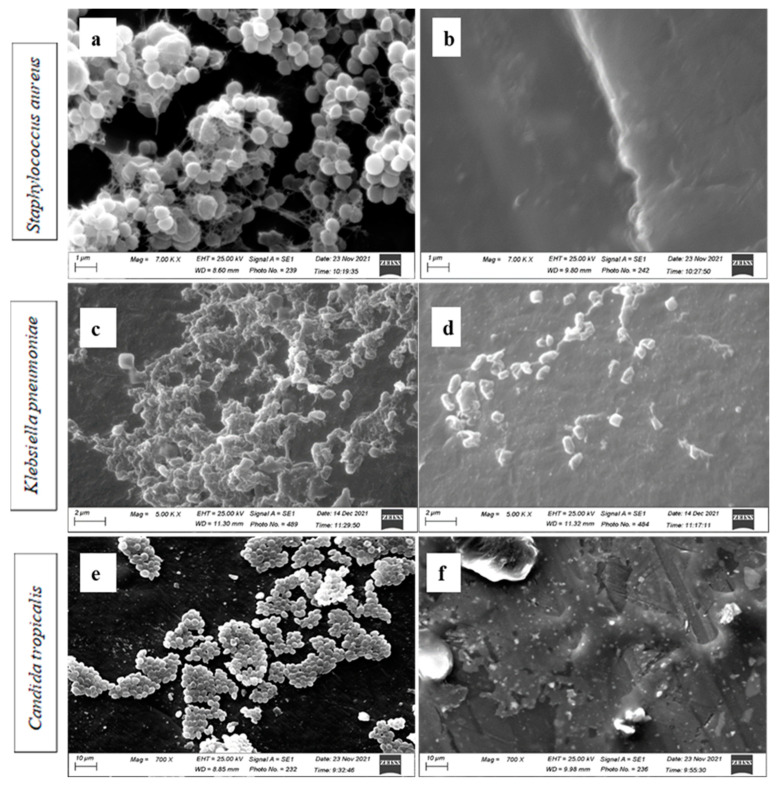
SEM micrographs of Staphylococcus aureus, Klebsiella pneumonia, and Candida tropicalis adherence to SR as shown in (**a**,**c**,**e**) and non-irradiated SR-g-AAc-ZnO at (**b**,**d**,**f**), respectively.

To visualize the inhibitory effect of non-irradiated SR-g-AAc-ZnO on the biofilm of the tested microbes, the SEM showed the disappearance of the accumulation of biofilm microbial cells on the surface of SR for Gram-positive *S. aureus* at 7000×, as shown in Figure 7a,b. Similarly, for Gram-negative *K. pneumoniae* at 5000× (Figure 7c) and the yeast *C. tropicalis* at 700× (Figure 7e), there were no or some separated cells on the surface of SR-g-AAc-ZnO, which may be planktonic (Figure 7d,f).

### 3.10. Effect of SR-g-AAc-ZnO on Gene Expression of Biofilm-Associated Genes in P. aeruginosa

Next, we aimed to study the effect of the non-irradiated SR-g-AAc-ZnO on the expression of selected biofilm-associated genes to understand the possible mechanism of action. Since the *P*. *aeruginosa* clinical isolate was the only XDR isolate (Table S6) among the strong biofilm producer, it had high MIC (Table 3). We challenged the modified silicone, and it reduced *P. aeruginosa* biofilm formation at sub-MIC (Figure 1) in addition to the complete inhibition of growth (Table 5). 

**Figure 8:**
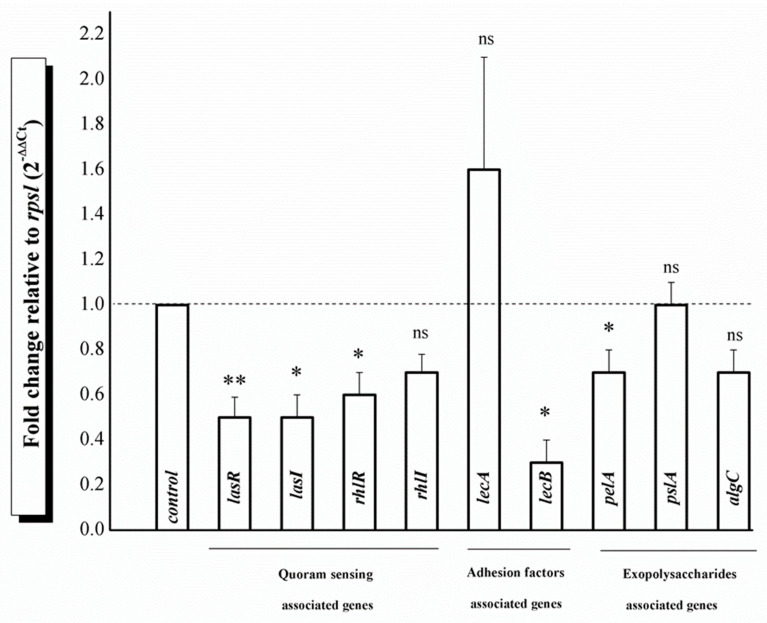
Effect of modified silicone surface on gene expression levels of biofilm-associated genes in *P. aeruginosa.* Fold change in gene expression of adhered *P. aeruginosa* on SR-g-AAc-ZnO relative to control SR determined using real-time RT-PCR. Fold change calculated using the ΔΔCt method with the *rpsl* gene as a housekeeping gene. The statistical significance (ns, not statistically significant; *, *p* < 0.05; **, *p* < 0.01) relative to the SR surface was calculated using a Student’s *t*-test.

Comparing the gene expression of biofilm-associated genes of *P. aeruginosa* under the modified treated silicone surface showed the downregulation of the expression levels of all tested genes except the *lecA* and *pslA* (Figure 8). The modified treated silicone surface (SR-g-AAc-ZnO) significantly downregulated the quorum sensing genes *lasR*, *lasI*, and *rhlR* by 2, 2, and 1.6-fold (*p*-value < 0.05), respectively. The modified treated silicone surface (SR-g-AAc-ZnO) also significantly down-regulated the *lecB* gene associated with bacterial adhesion by 3.3-fold at a *p*-value < 0.05. A significant fold change decrease of the *pelA* gene associated with polysaccharide production by 1.4-fold at *p*-value < 0.05 was observed. Although non-significant, there was an observed fold change decreased for the quorum sensing gene *rhlI* and the polysaccharide producing *algC* by 1.4-fold (*p*-value = 0.057 and *p*-value = 0.08, respectively). A non-statistically significant up-regulation of the *lecA* gene by 1.6-fold (*p*-value = 0.3) and no change in the *pslA* gene (*p*-value = 0.8) were seen (Figure 8). Data represent the mean fold change ± SD of two biological replicas, and a statistical analysis was performed using a Student’s t-test. The significance of the mean fold change was defined as * *p* < 0.05, ** *p* < 0.01. 

## 4. Discussion

Hospital-acquired infections (HAI) associated with the routine use of various biomaterials, such as urinary catheters, represent a significant health problem complicated by the worldwide rise in antimicrobial-resistant biofilm-forming bacteria. Generally, bacterial pathogens tend to aggregate and colonize on inert or biological interfaces and form biofilms rather than existing in a planktonic state. Biofilms are functional aggregates of bacteria shielded in extracellular polymeric substances (EPS) [4]. The negatively charged biofilm components and the highly viscous compact EPS structures protect the cells within it from exposure to innate immune defense and prevent the antibiotics from penetration, leading to the failure of biofilm infection treatment [4]. Biofilm formation on urinary catheters exhibits adverse effects both locally and systemically [8], which increase morbidity and mortality. Therefore, there is an urgent need for antimicrobial alternatives that are cost-effective for combating biofilms in catheter-associated HAI. 

Biomedical researchers began to reuse inorganic metals as antibiotic alternatives that produce distinctive and different forms of microbial cell injuries due to oxidative stress, protein malfunction, or membrane damage [69]. Several systems have been modified with inorganic nanoparticles for antimicrobial purposes, which are superior to conventional organic antibiotics since they are multifunctional and cost-effective materials that are stable for long-term storage due to their extended shelf-life and resistance to severe conditions, such as high temperature and sterilization. Polycarbonate medical devices have been modified with selenium nanoparticles to prevent microbial biofilm formation, as reported in [62]. Catheters have been modified by iron oxide nanoparticles [70] and magnesium fluoride nanoparticles [71] to prevent biofilm formation. Moreover, Zn-CuO was used in contact lens nanocoatings as an antibacterial [61]. Others used ZnO and CuO NPs on coating tooth models to inhibit *Streptococcus mutans* [13]. Applerot et al. [7] coated glass with ZnO NPs that inhibit bacterial biofilm formation and increased antibiotic susceptibility. In addition, ZnO NPs were used to modify textile surfaces such as cotton fibers for antibacterial finishing [57,72]. In the current study, we aimed to synthesize and use a modified silicone catheter with immobilized ZnO NPs as an integrated coating of urinary catheters.

Seventy-four clinical isolates were recovered and characterized as clinical isolates associated with silicone urinary catheters. Of those 74 clinical isolates, ten were found to be strong biofilm forming (Tables S1 and S2). Six of those ten-strong biofilm-forming isolates were chosen and identified morphologically, biochemically, and molecularly. These six isolates were used for the rest of the study as a representative sample of Gram-negative bacilli, Gram-positive cocci, and yeast (Table 2). These isolates were tested for the antimicrobial and anti-biofilm activity of both our in-house synthesized ZnO NPs and the modified silicone. ZnO NPs were prepared using the solvothermal synthesis method, ensuring the synthesis of nanoparticles with uniform spherical shapes without any dispersants [42]. Based on XRD results, synthesized ZnO NPs had a high purity of the hexagonal crystal structure judging by the JCPDS card no. 79-0207 of standard ZnO. Applying the Scherrer equation, as reported in [73], the crystalline nanoparticles’ size was calculated to be 10.3 nm. Moreover, TEM findings revealed a consistent shape that appeared to be approximately hexagonal or spherical. In addition, TEM results showed an average particle size of 14 nm, which is consistent with the XRD results. These results are per Mayekar et al. [74] and Šarić et al. [75], who examined changes in the particle form with different precursors and showed that using zinc acetate as a salt precursor ends with ZnO NPs in a spherical shape or by using ethanol as a solvent.

Synthesized ZnO NPs showed substantial broad-spectrum antimicrobial and anti-biofilm activities on both Gram-negative and positive clinical isolates and yeast isolates. ZnO NPs showed superior antimicrobial activity (MIC = 0.25 mg/mL) against *S. aureus* to those previously reported [76]. Although ZnO NPs inhibited *P. aeruginosa* isolate growth, its MIC was the highest, possibly due to the high antibiotic resistance of *P. aeruginosa*, as revealed by AST as XDR (Table S6). In addition, the microbial ability to adhere and form biofilm was inhibited after challenging with ZnO NPs at sub-MIC, where a notable decrease in the optical densities was observed (Figure 1 and Table S7). According to Stepanović et al. [46], comparing the resulting ODs with the ODc, we can conclude that the strong ability of all examined microbial isolates was suppressed.

One of the mechanisms responsible for antibacterial action most extensively documented in the literature is the formation of reactive oxygen species (ROS) by ZnO NPs. The production of ROS is due to the interaction between ZnO NPs and water molecules adsorbed on the NPs’ surface following activation by ultraviolet or visible light [11,12]. The photocatalytic activation of ZnO NPs leads to the movement of electrons from the valence to the conductive band, triggering a series of photoreactions [12] that produce different ROS. ROS as superoxide radical anions (˙O_2_^−^), hydroxyl radicals (˙OH), hydrogen peroxide (H_2_O_2_) and singlet oxygen (^1^O_2_) radicals can permanently damage bacterial membranes, DNA, and mitochondria, resulting in bacterial death [26]. Consequently, the antibacterial activity of ZnO NPs was enhanced with increased ROS generation following UV light exposure, as determined by Raghupathi and co-workers [77]. Another mechanism that can kill bacteria is the release of Zn^2+^ ions from the partial dissolution of ZnO NPs in solution [7,12], which can enter the bacterial cell and interact with the thiol groups of respiratory enzymes, blocking them and promoting the creation of more ROS [78,79]. In addition, Zinc ions can interact with the thiol groups of the proteins present on the bacterial cell surface responsible for adhesion and colonization [27]. ZnO NPs inactivate these surface proteins and change the membrane permeability, leading to the leakage of nucleic acid from the bacterial cells, thereby defining it as the most probable mechanism for ZnO NPs’ anti-biofilm activity [28]. Pati et al. [80] showed that ZnO NPs could compromise bacterial cell membrane integrity, lower cell surface hydrophobicity, and suppress the transcription of oxidative stress-resistance genes in bacteria. This antimicrobial activity of ZnO is more prominent in the nanoparticles range, as decreasing the particle size increases the surface area, so there are more sites for interaction with the microbial cells, as discussed by Nazoori and Kariminik [81]. 

Direct radiation-induced graft copolymerization of a poly-acrylic acid onto silicone was accomplished as reported in [55,56,82] and confirmed using FTIR and SEM depending on [34,83]. The grafting yield increased by increasing the radiation dose and the monomer concentration, as discussed by [34]. However, at a grafting yield percentage greater than 20, a whitish appearance and greater rigidity of silicone was observed, as previously reported by Magaña et al. [82]. Acrylic acid is one of the most used monomers grafted onto the surface of different polymeric matrices by this grafting technique. Adding hydrophilic functional groups of PAAc to hydrophobic polymers has been widely used to develop new systems with potential antibacterial applications and better biocompatibility [55]. For example, Vazquez-Gonzalez et al. [34] modified silicone with poly-methacrylic acid to add antiseptic drugs that prevented the in vitro growth of *Staphylococcus aureus.* Furthermore, Romero-Fierro et al. [84] modified cotton gauze with poly-acrylic acid to be modified with antimicrobial drugs, working as a delivery system that effectively treats skin wound infections. In addition, poly-acrylic acid was used as a nanoplatform for antimicrobial applications [85]. Cabana et al. [9] functionalized silicone with poly-acrylic acid nano-brushes to immobilize gold nanoparticles for photothermal antimicrobial therapy. Ni et al. [86] studied the antimicrobial activity of synthesized poly-acrylic acid-modified silver nanoparticles. In addition, Shaik et al. [87] reported a broad-spectrum antimicrobial activity of poly-acrylic acid-zinc composites. Others used poly(di(ethylene glycol)methyl ether methacrylate)—(POEGMA188) as well as poly(4-vinylpyridine)—(P4VP) based silver nanocomposite coatings attached to a glass surface, which exhibited antibacterial properties against Gram-positive *Staphylococcus aureus* ATCC 25923 and Gram-negative *Escherichia coli* ATCC 25922 [88,89]. 

Poly-acrylic acid grafted silicone was modified by immobilizing ZnO NPs using a rapid and low-cost in situ inter-matrix synthesis method [57]. First, the carboxyl groups in the grafted PAAc chains are activated by an alkali treatment. The pH is considered to be the crucial point at which the PAAc chains abruptly change from hydrophilic (extended state) to hydrophobic (collapsed state) [34]. In an acidic reaction solution, the long PAA chains are shrunken [9], as the COOH groups of PAAc are not ionized (collapsed state) and may form hydrogen bonds between polymeric chains, reducing exposure to the reaction solution. However, the ionization of COOH groups caused by alkali treatment (pH ˃ 6) creates repulsions within the PAAc chains, resulting in an extended, swollen configuration. Thus, the carboxylic acid groups in PAAc will be more accessible for ionic interactions with oppositely charged cationic zinc ions [34]. The monitoring of the FTIR of grafted silicone during ZnO NPs immobilization was according to Ellerbrock and Gerke [90], in which the absorption band of the characteristic carbonyl peak shifted to a lower wavenumber due to carboxylate formation as a result of ionization (Figure 4).

The grafted silicone was modified by immobilized ZnO NPs homogenously distributed over the treated silicone sample with no contaminants, as investigated by SEM-EDX analysis, confirming the efficient modification [72]. Modifying silicone by poly-acrylic acid grafting and immobilizing f ZnO NPs was further confirmed by thermogravimetric analysis. Comparing the thermogram of control silicone (SR), which has thermal stability [91], with the grafted silicone, two decomposition steps were observed before silicone degradation. This can be ascribed to the poly-acrylic acid degradation, as described by [92], which confirms the effective grafting of silicone as explained in [34]. After the immobilization of ZnO NPs, a slowdown in the decomposition rate of poly-acrylic acid was observed, rendering the modified silicone with better thermal stability.

The modified silicone catheter showed good cytocompatibility towards normal epithelial cells (OEC), as the viability of cells exposed to the modified silicone extracts was above 98% compared to the negative control and the extract of untreated silicone as well. Shalom et al. [93] indicated potential cytotoxicity by a reduction in cell viability below 70% of the level observed in the negative control (DMEM growth medium) treatment. Our observed good cytocompatibility could be ascribed to the high biocompatibility of poly-acrylic acid grafted on the modified silicone [9]. Previous studies using several toxicological approaches showed that ZnO NPs are bactericidal but non-toxic to human cells [14,15,16]. Consequently, ZnO can be used as a suitable additive for surfaces exposed to the human body [72].

Modified ZnO NPs grafted silicone inhibited the adherence of both tested Gram-positive and negative microbes to varying extents after three days of exposure. These findings can be attributed to the highly hydrated polymer brush-coating (PAAc), which allowed for minimal contact between the microorganisms and the underlying surface [94]. Therefore, microorganisms continue to grow as planktonic organisms rather than forming a biofilm. Furthermore, the formation of ROS and surface protein inactivation by ZnO NPs immobilized on the grafted silicone surface exhibit anti-biofilm action [7]. However, the adherence of *C. tropicalis* was not significantly affected by the modified ZnO NPs grafted silicone. This might be attributed to the strong upregulation of adhesins on the cell wall in the biofilm state [95]. Since the antibiofilm activity mechanism of ZnO NPs is mainly ascribed to surface protein inactivation and adhesins are upregulated in *C. tropicalis*, this upregulation might be counteracting the ZnO NPs effect. However, the exact reason for the little to no effect of modified ZnO NPs grafted silicone on *C. tropicalis* is unknown. In addition, it was observed that the irradiated modified silicone gave better antibiofilm activity toward *K. pneumonia* than did the non-irradiated one. This can be rationalized as the gamma irradiation enhancing the level of binding between the immobilized ZnO NPs and the grafted silicone due to the oxygen deficiency or defects created in the interface between ZnO and the grafted silicone after irradiation [96].

The anti-adherence activity of the modified silicone surface was demonstrated with a significant decrease (*p* < 0.05) in the expression of biofilm-associated genes in a selected *P. aeruginosa* clinical isolate. A significant decrease (*p* < 0.05) in the relative expression of quorum sensing (QS)-associated genes (*lasR*, *lasI*, and *rhlR*), *lecB* gene associated with adhesion, and *pelA* gene associated with exopolysaccharides in adhered cells on modified silicone compared to non-treated silicone was observed. We chose to test the effect of the modified silicone on the *P. aeruginosa* clinical isolate as a strong biofilm-producing XDR isolate. Moreover, *P. aeruginosa* is a potent opportunistic bacterium responsible for many infections including urinary tract infections. Due to its QS systems and cell-density dependent signaling mechanism, *P. aeruginosa* develops biofilms on medically critical devices such as urinary catheters. The QS systems work by directing the production of various virulence agents and influencing the host immunological response [97]. *LasR*/*I* and *rhlR*/*I* are two principal QS systems that control virulence genes’ production in *P. aeruginosa*. The downregulation of these systems leads to the suppression of other virulence factors. Saleh et al. [98] reported that ZnO NPs significantly decreased the relative expression of the QS-genes *lasI*, *lasR*, *rhlI*, and *rhlR*. Adhesion factors are essential for the attachment of bacterial cells to the surfaces. In *P. aeruginosa* biofilms, adhesion factors such as lectins (lecA and lecB) play a vital role in adhesion and biofilm development [99]. Suppressing their encoding genes will significantly inhibit adherence. Moreover, the *pelA* gene is related to polysaccharide synthesis, a fundamental component of EPS that contributes to biofilm architecture. In this study, ZnO NPs modified silicone caused a significant downregulation of the *pelA* gene, consequently contributing to the blocking of EPS production by *P. aeruginosa*. This might be a strategy to overcome EPS barriers by passively enhancing the interactions of ZnO NPs modified silicone and biofilm through blocking EPS production, a strategy of nanoparticle action as reviewed in [4]. 

Our gene-expression analysis showed that treated silicone inhibited *P. aeruginosa* biofilm by repressing the QS, adhesion, and polysaccharide production systems’ activity. It was previously reported that ZnO NPs inhibit *P. aeruginosa* virulence genes’ expression and the QS system [98,99,100,101]. However, the exact mechanism of action still remains to be fully understood. Others studying silver (Ag) NPs’ effect on *P. aeruginosa* virulence factors, including biofilm formation, proposed a mechanism of how Ag NPs interrupt QS system activity. Singh et al. [102] proposed that in the absence of Ag NPs, the *P. aeruginosa* Las-Rhl QS system produces signaling molecules as N-acyl homoserine lactones (AHLs) that assess a predetermined cell density. If this predetermined cell density level is surpassed, the LuxR-AHLs complex initiates virulence and biofilm-associated gene expression. Singh et al. postulated that Ag NPs are internalized by *P. aeruginosa* cells interfering with the QS system by limiting LasIR-RhlIR-mediated AHLs synthesis. Ag NPs’ induced reduction of AHLs leads to the inhibition of the expression of target genes that encode virulence factors and biofilms. The molecular basis and complete mechanism of action of how nanoparticles impact QS and inhibit biofilm formation are currently being studied.

## 5. Conclusions

Our results collectively showed that gamma rays could easily functionalize the silicone catheter by grafting for the further modification by antimicrobial agents such as ZnO NPs. Modified ZnO NPs’ grafted silicone produced biocompatible catheters that inhibited the adherence of pathogenic microbes, rendering silicone catheters with self-anti-biofouling activity. Using these modified catheters in hospital settings would reduce HAI, morbidity, and mortality. Further studies are recommended to confirm the in vivo effectiveness of the modified silicone catheter in preventing biofilm formation and the potential use in hospital settings. 

## Figures and Tables

**Table 1:** List of primers used in the real-time PCR of selected biofilm-associated genes.

Gene		Gene ID	Sequence (5’-3’)	Reference
House-keeping gene	30S ribosomal protein S12 (*rpsL*)	881709	AACTCGGCACTGCGTAAG	This study
			TGTGCTCTTGCAGGTTGT	
Quorum-sensing associated genes	Transcriptional regulator LasR (*lasR*)	881789	CTGTGGATGCTCAAGGACTAC	This study
			CCACTGCAACACTTCCTTCT	
	Acyl-homoserine-lactone synthase (*lasI*)	881777	GGCTGGGACGTTAGTGTCAT	[66]
			AAAACCTGGGCTTCAGGAGT	
	Transcriptional regulator RhlR (*rhlR*)	878968	GGCTTCGATTACTACGCCTATG	This study
			CCGTAGTTCTGCATCTGGTATC	
	Acyl-homoserine-lactone synthase (*rhlI*)	878967	GCAGCTGGCGATGAAGATA	This study
			GCCGTTGCGAACGAAATAG	
Adhesion factors associated genes	PA-I galactophilic lectin (*lecA*)	882335	CACCATTGTGTTTCCTGGCGTTCA	[67]
			AGAAGGCAACGTCGACTCGTTGAT	
	Fucose-binding lectin PA-IIL (*lecB*)	882528	AGACAGCGTAACAATCGAACGAGC	[67]
			AGGACGCATCGTTCAGCCAATCTA	
Exopolysaccharide-associated genes	Hypothetical protein PA3064 (*pelA*)	878833	CCTTCAGCCATCCGTTCTTCT	[68]
			TCGCGTACGAAGTCGACCTT	
	Biofilm formation protein PslA (*pslA*)	879717	TGATCTTCTGGTTCACCGGC	This study
			GGTACATGCCGCGTTTCATC	
	Phosphomannomutase (*algC*)	879406	GGCAGATCCGTTGTTCCAGA	This study
			TCATGATGGGGGTTTGCTCC	

**Table 2:** Molecular identification of the selected six strong biofilm-forming isolates using *16S rRNA* sequencing.

Clinical Isolate Number	Identification	% Identity	Query Coverage	e-Value	Accession No.
#26	*Pseudomonas aeruginosa* DSM 50071	99.23%	98%	0.0	NR117678.1
#36	*Escherichia coli* NBRC 102203	95.38%	100%	1 × 10^−73^	NR114042.1
#56	*Alcaligenes faecalis* NBRC 13111	97.85%	100%	0.0	NR113606.1
#55	*Klebsiella pneumonia* DSM 30104	99.53%	100%	0.0	NR117683.1
#35	*Staphylococcus aureus* S33 R	99.66%	100%	0.0	NR037007.2
#06	*Candida tropicalis* YDP38	100%	100%	0.0	MT341912.1

**Table 3:** Minimum inhibitory concentration of ZnO nanoparticles (ZnO NPs) against the isolated strong biofilm selected isolates with reference to standard microbes.

Microbe		MIC of ZnO NPs (mg/mL)
**Clinical Isolates**	*P. aeruginosa*	16
	*K. pneumoniae*	1.0
	*E. coli*	0.5
	*A. faecalis*	1.0
	*S. aureus*	0.25
	*C. tropicalis*	8.0
**Standard Strains**	*P. aeruginosa (*PAO1)	16.0
	*K. pneumoniae* (2S11122)	0.25
	*E. coli (*K12MG1655)	1.0
	*S. aureus (*MRSA—N315)	2.0
	*S. aureus (*MSSA—RN6319)	0.25

**Table 4:** Cell viability % of normal human oral epithelial cells (OEC) with extracts of SR and SR-g-AAc-ZnO segments.

Extract Name	Mean OD ± SD (540 nm)	Cell Viability %
Extract of SR	1.724 ± 0.009	99.36
Extract of SR-g-AA-ZnO	1.713 ± 0.020	98.71
Two-fold diluted extract of SR-g-AA-ZnO (1:2)	1.729 ± 0.003	99.63
Four-fold diluted of extract of SR-g-AA-ZnO (1:4)	1.720 ± 0.009	99.13
Negative control	1.740 ± 0.041	100

**Table 5:** Mean log viable count of adhered cells on 5 × 5 mm silicone segments after 72 h incubation.

	Segment Used	Average Viable Count (log CFU/Segment ± SD) ^1^		
Microbial Isolate		SR	Non-Irradiated SR-g-AAc-ZnO	Irradiated SR-g-AAc-ZnO
*P. aeruginosa*		4.5 ± 0.20	- ^2^	-
*K. pneumonia*		4.7 ± 0.10	3.4 ± 0.10	2.0 ± 0.04
*E. coli*		3.0 ± 0.00	-	-
*A. faecalis*		3.5 ± 0.02	2.9 ± 0.05	2.9 ± 0.02
*S. aureus*		4.7 ± 0.10	-	-
*C. tropicalis*		4.6 ± 0.25	4.2 ± 0.06	4.1 ± 0.17

^1^ mean ± SD of at least three biological replica; comparison using one-way ANOVA followed by post hoc Dunnett’s test, and the significance was defined as *p* < 0.05, ^2^ non-detected.

## Data Availability

The data presented in this study are available in the article and supplementary material files.

## Supplementary Materials

The following supporting information can be downloaded at: https://www.mdpi.com/article/10.3390/microorganisms11040913/s1, Table S1: Preliminary identification of microbial isolates and their biofilm forming ability; Table S2. Summary of the classification of microbial isolates recovered from urinary silicone catheters according to their growth on culture media, Gram-staining reaction, and degree of biofilm formation; Table S3: Biochemical identification of selected strong-biofilm forming clinical isolates using the VITEK 2 compact system; Table S4: Antimicrobial susceptibility testing of Gram-negative strong-biofilm forming clinical isolates and their interpretation; Table S5: Antimicrobial susceptibility testing of Gram-positive strong-biofilm forming clinical isolate and its interpretation; Table S6. Summary of the classification of the strong biofilm forming isolates according to the interim standard definitions for acquired resistance; Figure S1: X-ray diffraction analysis patterns of the synthesized ZnO NPs relative to the standard ZnO NPs; Figure S2: Transmission electron microscopy image of the synthesized ZnO NPs; Figure S3: Scanning electron microscopy (SEM) microphotograph of SR and SR-g-AAc.
