# Supplementary material for: Inhibition of Adherence and Biofilm Formation of Pseudomonas aeruginosa by Immobilized ZnO Nanoparticles on Silicone Urinary Catheter Grafted by Gamma Irradiation
Source: Microorganisms. 2023 Mar 31;11(4):913. doi: 10.3390/microorganisms11040913 (PMC10142706; doi:10.3390/microorganisms11040913)
Supplement: Supplementary file 1 [file microorganisms-11-00913-s001.zip › microorganisms-2314023-supplementary.pdf]

Table S1: Preliminary identification of microbial isolates and their biofilm forming ability

Isolate number	Categorization of Isolates Based on Culturing and Gram Staining[1]	Biofilm Formation (Average Optical Density at 630 nm)[2,3]	Classification of Biofilm Production[4]
1	G-ve LF	0.346	++
2	G-ve LF	0.170	+
3	G-ve NLF	0.155	0
4	G-ve LF	0.291	+
5	G-ve NLF	0.203	+
**6***	**yeast**	**0.870**	**+++**
7	yeast	0.490	++
**8***	**G-ve LF**	**0.953**	**+++**
9	yeast	0.432	++
10	yeast	0.501	++
11	G-ve LF	0.568	++
12	G-ve LF	0.209	+
13	G-ve LF	0.153	0
14	G-ve NLF	0.175	+
15	yeast	0.214	+
16	yeast	0.370	++
17	G-ve LF	0.180	+
18	G-ve LF	0.350	++
19	G+ve cocci	0.439	++
20	yeast	0.321	++
21	G-ve LF	0.388	++
22	G-ve LF	0.135	0
23	G-ve NLF	0.470	++
24	G-ve NLF	0.599	++
25	G-ve LF	0.165	+
**26***	**G-ve NLF**	**0.947**	**+++**
27	yeast	0.468	++
28	yeast	0.193	+
29	G-ve LF	0.229	+
30	yeast	0.395	++
31	G-ve LF	0.131	0
32	G-ve LF	0.270	+
33	G-ve LF	0.098	0
34	G+ve cocci	0.114	0
**35***	**G+ve cocci**	**0.751**	**+++**
**36***	**G-ve LF**	**0.702**	**+++**
37	yeast	0.207	+

[1] culturing on nutrient agar, MacConkey no. 3 and Sabaroud agar followed by Gram staining, G+ve: Gram-positive, G-ve: Gram-negative, LF: lactose fermenters, NLF: non-lactose fermenters. [2]Average Blank = 0.125; [3]Optical density cut-off value (ODc) = 0.158; [4] 0: non-biofilm forming, +: weak, ++: moderate, and +++: strong-biofilm producing. *selected strong-biofilm producing isolated.

Table S1: continued

Isolate number	Categorization of Isolates Based on Culturing and Gram Staining[1]	Biofilm Formation (Average Optical Density at 630 nm)[2,3]	Classification of Biofilm Production[4]
38	G-ve LF	0.406	++
39	G-ve LF	0.356	++
**40***	**G-ve NLF**	**0.884**	**+++**
41	yeast	0.191	+
42	G+ve cocci	0.350	++
43	G-ve LF	0.145	0
44	G-ve LF	0.197	+
45	yeast	0.489	++
46	G-ve LF	0.149	0
47	G-ve LF	0.244	+
48	G+ve cocci	0.319	++
**49***	**G-ve LF**	**0.869**	**+++**
50	G+ve cocci	0.380	++
51	G-ve LF	0.310	+
52	G-ve NLF	0.159	+
53	yeast	0.174	+
54	G-ve LF	0.138	0
**55***	**G-ve LF**	**0.859**	**+++**
**56***	**G-ve NLF**	**0.801**	**+++**
57	yeast	0.405	++
58	yeast	0.233	+
59	G+ve cocci	0.490	++
60	G+ve cocci	0.260	+
**61***	**G-ve NLF**	**1.069**	**+++**
62	G-ve LF	0.350	++
63	G-ve LF	0.449	++
64	G-ve NLF	0.576	++
65	yeast	0.346	++
66	G-ve NLF	0.190	+
67	G+ve cocci	0.400	++
68	G-ve LF	0.317	++
69	G-ve NLF	0.163	+
70	G-ve NLF	0.360	++
71	yeast	0.178	+
72	G-ve LF	0.174	+
73	G-ve LF	0.12	0
74	G+ve cocci	0.37	++

[1] culturing on nutrient agar, MacConkey no. 3 and Sabaroud agar followed by Gram staining, G+ve: Gram-positive, G-ve: Gram-negative, LF: lactose fermenters, NLF: non-lactose fermenters. [2]Average Blank = 0.125; [3]Optical density cut-off value (ODc) = 0.158; [4] 0: non-biofilm forming, +: weak, ++: moderate, and +++: strong-biofilm producing. *selected strong-biofilm producing isolated.

Table S2. Summary of the classification of microbial isolates recovered from urinary silicone catheters according to their growth on culture media, Gram-staining reaction, and degree of biofilm formation

Biofilm production / Microbe	Strong biofilm	Moderate biofilm	Weak biofilm	Non-biofilm	Total
Gram Negative Bacilli	8	13	16	9	46
Gram Positive Cocci	1	7	1	1	10
Yeast	1	10	7	0	18
Total	10	30	24	10	74

Table S3: Biochemical Identification of Selected Strong-biofilm forming Clinical Isolates using VITEK 2 Compact system

**Type**	**Microbial Type**	**Isolate Number**	**Strain Identification by Vitek2 compact**	**Bionumber**
**Gram-negative bacilli**	Lactose Fermenters	8	*Klebsiella pneumoniae*	6607730773565010
		49		6607734753564010
		55		6667735753765011
		36	*Escherichia coli*	0405610554566211
	Non-Lactose Fermenters	26	*Pseudomonas aeruginosa*	0003043003500252
		40		0003453143500210
		61		0003453143500210
		56	*Alcaligenes faecalis*	0000001103500042
**Gram-positive cocci**	cocci	35	*Staphylococcus aureus*	3063153303520050
**Fungal isolates**	yeast	6	*Candida tropicalis*	6627734553566010

Table S4: Antimicrobial Susceptibility Testing of Gram negative Strong-biofilm forming Clinical Isolates and their Interpretation[1]

Clinical Isolate / Antibiotic tested	*E. coli*	*A. faecalis*	*K. pneumonia*			*P. aeruginosa*		
	Isolate 36	Isolate 56	Isolate 55	Isolate 49	Isolate 8	Isolate 26	Isolate 40	Isolate 61
Beta-lactams								
Ampicillin	≥ 32 / R[2]	-	≥ 32 / R	≥ 32 / R	≥ 32 / R	-	-	-
Ampicillin/Sulbactam	≥ 32 / R	-	≥ 32 / R	≥ 32 / R	≥ 32 / R	-	-	-
Piperacillin/Tazobactam	≥128 / R	≥ 128 / R	≥ 128 / R	16 / S[3]	16 / S	≥ 128 / R	≥ 128 / R	≥ 128 / R
Cefazolin	≥ 64 / R	16 / I[4]	≥ 64 / R	≥ 64 / R	≥ 64 / R	≥ 64 / R	≥ 64 / R	≥ 64 / R
Cefoxitin	≥ 64 / R	-	≥ 64 / R	≥ 64 / R	≥ 64 / R		-	-
Ceftazidime	32 / R	4 / S	≥ 64 / R	8 / I	8 / I	≥ 64 / R	32 / R	32 / R
Ceftriaxone	≥ 64 / R	≤ 1 / S	≥64 / R	≤ 1 / S	≤ 1 / S	-	-	-
Cefepime	≥ 64 / R	8 / S	≥ 64 / R	≤ 1 / S	≤ 1 / S	≥ 64 / R	≥ 64 / R	≥ 64 / R
Meropenem	≤ 0.25 / S	≤ 0.25 / S	≤ 0.25 / S	≤ 0.25 / S	≤ 0.25 / S	≤ 0.25 / S	≤ 0.25 / S	≤ 0.25 / S
ESBL	NEG	-	NEG	NEG	NEG	-	-	-
Aminoglycoside								
Amikacin	≤ 2 / S	4 / S	4 / S	≤ 2 / S	≤ 2 / S	4 / S	≤ 2 / S	≤ 2 / S
Gentamicin	≤ 1 / S	2 / S	≥ 16 / R	≤ 1 / S	≤ 1 / S	≥ 16 / R	≤ 1 / S	≤ 1 / S
Tobramycin	≤ 1 / S	2 / S	≥ 16 / R	≤ 1 / S	≤ 1 / S	≥ 16 / R	≤ 1 / S	≤ 1 / S
Quinolones								
Ciprofloxacin	≤ 0.25 / S	1 / S	≥ 4 / R	0.5 / S	0.5 / S	≥ 4 / R	≤ 0.25 / S	≤ 0.25 / S
Levofloxacin	1 / S	1 / S	≥ 8 / R	1 / S	1 / S	≥ 8 / R	1 / S	1 / S
Nitrofurantoin	≥ 512 / R	-	≤ 16 / S	64 / I	64 / I	-	-	-
Folate inhibitors								
Trimethoprim/Sulfamethoxazole	≥ 320 / R	≤ 20 / S	≥ 320 / R	≤ 20 / S	≤ 20 / S	-	-	-

[1] interpretation according to Performance Standards for Antimicrobial Susceptibility Testing. 32nd ed. CLSI supplement M100. Clinical and Laboratory Standards Institute; 2022.[2] R: resistant, [3]S: sensitive, [4]I: intermediate.

Table S5: Antimicrobial Susceptibility Testing of Gram positive Strong-biofilm forming Clinical Isolate and its Interpretation[1]

**Antibiotics tested**	***Staphylococcus aureus*** **(Isolate 35)**	
	MIC µg/mL	**Interpretation**
Beta-lactams		
Benzylpenicillin	≤ 0.03	S
Oxacillin	1	S
Cefoxitin Screen	Positive	
Aminoglycoside		
Gentamicin	≤ 0.5	S
Quinolones		
Ciprofloxacin	≤ 0.5	S
Levofloxacin	0.25	S
Moxifloxacin	≤ 0.25	S
Macrolides		
Erythromycin	0.5	S
Clindamycin	≤ 0.25	S
Inducible Clindamycin Resistance	Negative	
Streptogramin		
Quinupristin/Dalfopristin	0.5	S
Oxazolidinones		
Linezolid	4	S
Glycopeptide		
Vancomycin	1	S
Tetracycline		
Tetracycline	≤ 1	S
Glycylcycline		
Tigecycline	≤ 0.12	S
Others		
Nitrofurantoin	≤ 16	S
Antimycobacterials		
Rifampicin	≤ 0.5	S
Folate inhibitors		
Trimethoprim/Sulfamethoxazole	≤ 10	S

[1] interpretation according to Performance Standards for Antimicrobial Susceptibility Testing. 32nd ed. CLSI supplement M100. Clinical and Laboratory Standards Institute; 2022.

Table S6. Summary of the classification of the strong biofilm forming isolates according to the interim standard definitions for acquired resistance

Strong Biofilm-producing clinical isolates		Classification of Antibiotics Susceptibility
Klebsiella pneumoniae	#08	S[1]
	#49	S
	#55*	MDR[2]
Pseudomonas aeruginosa	#61	S
	#40	S
	#26*	XDR[3]
Alcaligenes faecalis	#56*	S
Escherichia coli	#36*	MDR
Staphylococcus aureus	#35*	S

* Molecularly identified isolates, [1] S: susceptible, [2]Multidrug-resistant, [3] extensively drug-resistant.

Table S7. Effect of ZnO NPs at subMICs concentrations on growth and biofilm formation by selected isolates

**Microbe** \ **ZnO NPs (mg/mL)**	**Control**	**0.062**	**0.125**	**0.25**	**0.5**	**1**	
P. aeruginosa	+	+	+	+	+	+	Growth[1]
	0.947 ± 0.1	0.306 ± 0.091	0.257 ± 0.095	0.189 ± 0.007	0.171± 0.041	0.165 ± 0.067	OD630
K. pneumoniae	+	+	+	+	+	+	Growth
	0.859 ± 0.1	0.225 ± 0.009	0.352 ± 0.026	0.448 ± 0.007	0.488 ± 0.117	0.478 ± 0.120	OD630
E. coli	+	+	+	+	-	-	Growth
	0.702 ± 0.1	0.104 ± 0.004	0.112 ± 0.004	0.109 ± 0.006	0.109 ± 0.011	0.111 ± 0.003	OD630
A. faecalis	+	+	+	+	+	-	Growth
	0.801 ± 0.1	0.306 ± 0.043	0.350 ± 0.084	0.218 ± 0.089	0.262 ± 0.091	0.198 ± 0.068	OD630
S. aureus	+	+	+	-	-	-	Growth
	0.751 ± 0.1	0.760 ± 0.115	0.406 ± 0.194	0.259 ± 0.131	0.163 ± 0.004	0.291 ± 0.079	OD630
C. tropicalis	+	+	+	+	+	+	Growth
	0.870 ± 0.1	0.259 ±0.016	0.251 ± 0.016	0.177 ± 0.053	0.149 ± 0.031	0.142 ± 0.026	OD630

1: growth either as + = growth or - = no growth observed

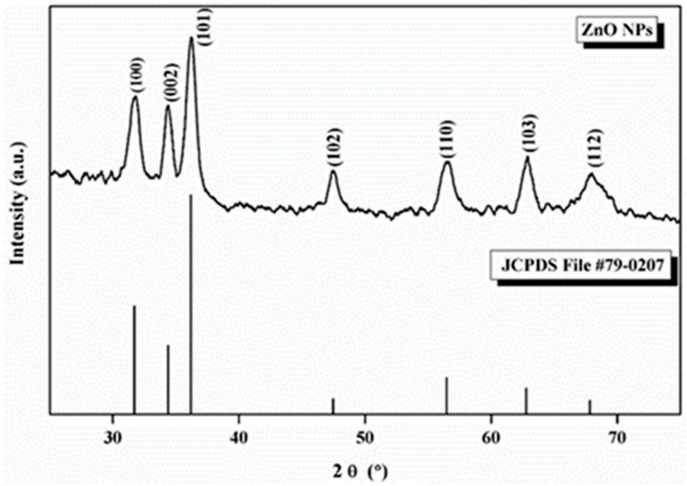

Figure S1. X-ray Diffraction Analysis patterns of the synthesized ZnO NPs relative to the standard ZnO NPs

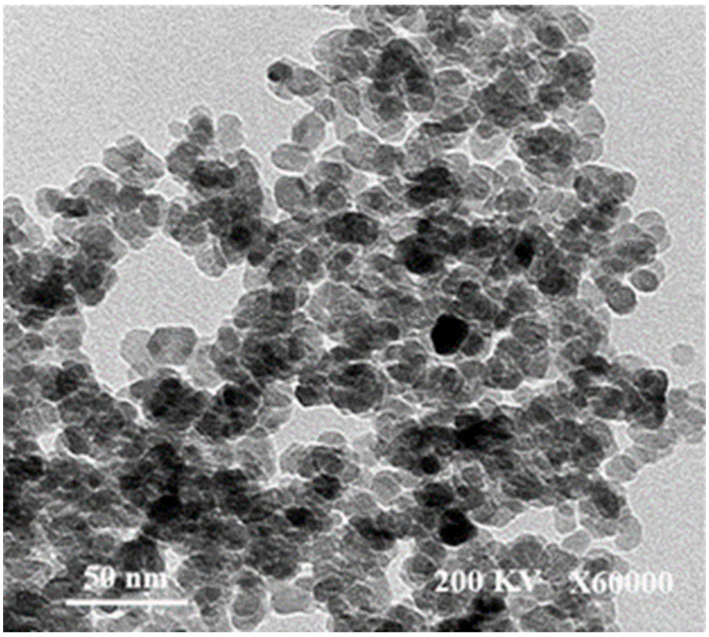

Figure S2. Transmission Electron Microscopy Image of the synthesized ZnO NPs

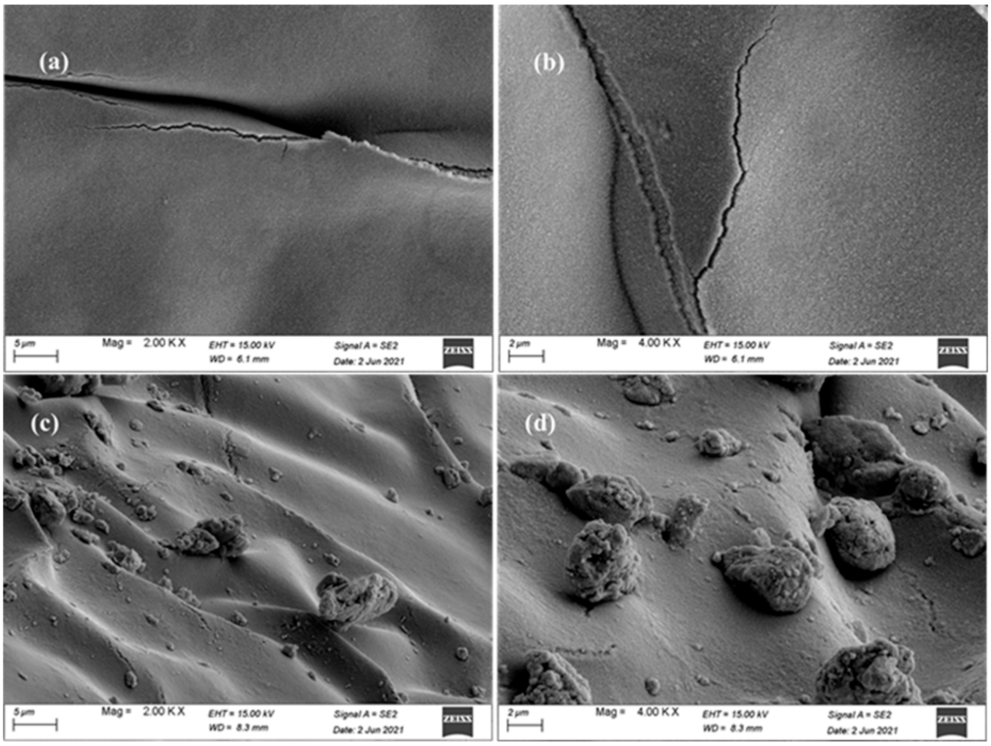

Figure S3. Scanning electron microscopy (SEM) microphotograph of SR and SR-g-AAc. **(a)** SR at at 2.00 KX and (v) at 4.00 KX, SR-g-AAc at (c) 2.00 KX and (d) 4.00 KX.
